# Health Inequity in Cervical Cancer Control Among Refugee Women in the United States by Country of Origin

**DOI:** 10.1089/heq.2020.0108

**Published:** 2021-03-04

**Authors:** Catherine E. Elmore, Jessica Keim-Malpass, Emma McKim Mitchell

**Affiliations:** ^1^ School of Nursing, University of Virginia, Charlottesville, Virginia, USA.; ^2^ Department of Acute and Specialty Care, School of Nursing, University of Virginia, Charlottesville, Virginia, USA.; ^3^ Department of Pediatrics, School of Medicine, University of Virginia, Charlottesville, Virginia, USA.; ^4^ Department of Family, Community and Mental Health Systems, School of Nursing, University of Virginia, Charlottesville, Virginia, USA.

**Keywords:** cancer screening, refugees, national health policy, health care disparities, women's health, international health

## Abstract

**Purpose:** To describe cervical cancer control practices from common countries of origin for women who resettle in the United States as refugees to highlight this persistent health inequity.

**Methods:** Describe presence/type of national cervical cancer screening program, screening coverage percentage, and human papillomavirus (HPV) vaccination program presence and coverage.

**Results:** Nine of 15 included countries screen opportunistically. Most do not use high-performing tests, and estimates of screening coverage were limited. Only one country offers HPV vaccination.

**Conclusion:** Countries of origin for refugee women may lack effective national cervical cancer control programs. To meet the World Health Organization (WHO)'s call to eliminate cervical cancer by 2030, focus on culturally tailored education, and continued research are paramount.

## Purpose

Worldwide cervical cancer continues to be the fourth leading cause of cancer incidence and death for women,^1^ and the burden is highest in low and middle income countries (LMICs).^1^ In the United States, foreign-born women are significantly more likely to have *never* been screened for cervical cancer, and are significantly more likely to be past due for cervical cancer screening (CCS).^2–5^ Many refugees entering the United States come from LMICs where screening programs for cervical cancer are inaccessible and/or under-resourced.^6,7^ Even where CCS programs exist, many refugees' countries of origin have no active invitation to screening, meaning they are opportunistic and do not track data using screening registries.^8^ We posit that women resettle in the United States with knowledge, attitudes, and beliefs about preventive care in general, and screening for cervical cancer in particular, which are informed by programs, and practices of their countries of origin.

We conducted a retrospective cohort study of women (*n*=525) who arrived in the United States as refugees and are current patients at a family medicine clinic to determine predictors of CCS adherence (Elmore et al. 2020 in preparation). Because factors related to countries of origin could be important predictors, we examined country-level data related to primary and secondary prevention of cervical cancer including human papillomavirus (HPV) vaccination and cervical screening programs. For our sample, the country-level data were skewed toward the absence of CCS programs or lacked representative population-level coverage estimates such that including these variables in statistical models would have been futile: the large majority of women came from countries without comprehensive cervical cancer control programs.

The aim of this short report is to describe country-level cervical cancer control programs from common countries of origin for women who resettle in the United States as refugees to highlight this persistent health inequity.

## Methods

We collected and tabulated descriptive data including (1) the presence (yes/no) and type (organized population-based screening vs. opportunistic screening) of a national CCS program; (2) the percentage of screening coverage of the population; (3) the type of screening being done (e.g., cytology, HPV genotyping, and visual inspection of the cervix with acetic acid [VIA]); and (4) the presence (yes/no) and percentage of HPV vaccination coverage.

The 10 most frequent countries of origin from our study sample (given in Table 1) and the 10 most frequent countries of origin for U.S. refugee admissions from fiscal year (FY) 2019^9^ (given in Table 2) were considered. Five countries overlap; therefore, data from 15 countries are used to examine how countries of origin may influence cervical cancer control for refugee populations resettled in the United States. We also consider four specific countries of origin (Afghanistan, Democratic Republic of Congo, Colombia, and El Salvador) as examples on either ends of the spectrum of cervical cancer control programs.

**Table 1.** Top 10 Countries of Origin for Our Sample^a^ (*n*=525)

Countries of origin (top 10)	Frequency	%	Cumulative %
Afghanistan	130	24.8	24.8
Bhutan^b^	109	20.8	45.6
Iraq	73	13.9	59.5
DR Congo	35	6.7	66.2
Burma	33	6.3	72.5
Nepal^b^	20	3.8	76.3
Syria	18	3.4	79.7
Colombia	11	2.1	81.8
Iran	9	1.7	83.5
Russia	9	1.7	85.2

^a^ Includes only women, 21+ years old.

^b^ Women who report country of origin as either Bhutan or Nepal are ethnically Bhutanese and have lived in refugee camps in Nepal for many years.

DR Congo=Democratic Republic of the Congo.

**Table 2.** Top 10 Countries of Origin for Refugee Resettlement in the United States Fiscal Year 2019^9^ (*n*=11,814)

Countries of origin (top 10)	Frequency^a^	%	Cumulative %
DR Congo^b^	2868	24.3	24.3
Burma^b^	2115	17.9	42.2
Ukraine	1927	16.3	58.5
Afghanistan^b^	604	5.1	63.6
Iraq^b^	537	4.5	68.1
Syria^b^	481	4.1	72.2
Eritrea	475	4.0	76.2
El Salvador	365	3.1	79.3
Moldova	364	3.1	82.4
Sudan	254	2.1	84.5

^a^ Number of individuals, includes all genders and ages.

^b^ Country overlaps with those in Table 1.

Data were obtained from:

(1) Review of most frequent countries of origin in our study sample (Table 1). This includes only females over the age of 21 years who are current attendees of a family medicine clinic (*n*=525), average years in the United States is 6.1 (range <1 to 20.5 years).
(2) Review of Worldwide Refugee Admissions Processing System data on most frequent countries of origin for FY 2019 (Table 2). These data include individuals of all genders and all ages (*n*=11,814), and provide a snapshot recent refugee resettlement trends in the United States.
(3) Review of data from HPV Information Centre.^8^ We examined “Human Papillomavirus and Related Diseases” full reports by countries of origin (last updated June 17, 2019). HPV Information Centre is a collaboration between Catalan Institute of Oncology (ICO) and International Agency for Research on Cancer (IARC) that compiles data from systematic review and meta-analysis of published literature and from official reports by the World Health Organization (WHO), the United Nations, The World Bank, and IARC's *Globocan* and *Cancer Incidence in Five Continents*.
(4) Review of data from Gavi: The Vaccine Alliance country hub website,^10^ examining whether or not the country was receiving Gavi support specifically for HPV vaccination.

This study uses publicly available country-level data and, therefore, no review by the institutional review board was required.

## Results

According to HPV Centre data, 9 of the 15 countries have CCS programs; of these, all are opportunistic, meaning there is no active program by which women are invited to be seen for screening (Table 3). Six national programs use cytology alone, one uses VIA alone, one uses cytology and VIA, and only one country (Colombia) uses either cytology, VIA, or HPV genotyping based on age.

**Table 3.** Comparison of Primary and Secondary Prevention Strategies for Cervical Cancer for 15 Countries of Origin of Refugees Who Resettle in the United States

Country of origin	National screening program	Type of national program	Percentage of screening coverage	Most widely used screening method	National HPV vaccination program percentage coverage	Receiving support from Gavi for HPV vaccination
United States	Yes	Opportunistic	80.7%^a^	Cytology/HPV	64.2% 44.5%^m^	No
Afghanistan	Yes	Opportunistic	—	Cytology	—	No
Bhutan	No	—	40.9%^d^	VIA^b^	—	No
Burma	No	—	—	VIA	—	No
Colombia	Yes	Opportunistic	69.9%^e^	Cytology/VIA/HPV^c^	85% 56%^^*^^	No
Congo, DR	No	—	—	—	—	No
El Salvador	Yes	Opportunistic	66.7%^f^	Cytology/VIA	—	No
Eritrea	No	—	—	—	—	No
Iran	Yes	Opportunistic	49.4%^g^	Cytology	—	No
Iraq	Yes	—	12.6%^h^	Unknown	—	No
Moldova	Yes	Opportunistic	30%^i^	Cytology	—	Yes
Nepal	Yes	Opportunistic	2.8%^j^	VIA	—	No
Russia	Yes	Opportunistic	72%^k^	Cytology	—	No
Sudan	No	—	—	VIA^b^	—	No
Syria	Yes	Opportunistic	—	Cytology	—	No
Ukraine	Yes	Opportunistic	73.7%^l^	Cytology	—	No

United States is included as reference country.

^^*^^2018 Females 15 years and older: 85% first dose and 56% full HPV vaccine series.

“—”indicates no data found or reported.

Data sources: HPV Centre Human Papillomavirus and Related Diseases Report for respective countries, version 17 June 2019; Gavi: The Vaccine Alliance country hub data; WHO.

^a^ Based on 2013 population-based survey, *n*=11,857, 21–65, within past 3 years.

^b^ Demonstration project only.

^c^ Method depends on screening age (years): 25–69 (cytology), 30–50 (VIA), 30–69 (HPV).

^d^ Based on 2012 population-based survey, *n*=13,852, age 20–59, ever screened.

^e^ Based on 2010 population-based survey, *n*=6339, age 18–69, every 1 year.

^f^ Based on 2003 population-based survey, *n*=8777, age 15–49, within past 2 years.

^g^ Based on single 2015 urban study, *n*=441, age 18–49, ever screened.

^h^ Based on single 2012 urban study, *n*=222, ≥20, ever screened.

^i^ Based on statistical modeling from 2013, ever screened.

^j^ Based on 2003 population-based survey, *n*=3486, age 25–64, every 3 years.

^k^ Based on 2012 population-based survey, *n*=unknown, age 14–55, every 3 years.

^l^ Based on 2003 population-based survey, *n*=1007, age 25–64, every 3 years.

^m^ 1994 birth cohort has the highest percentage coverage: 64.2% for one dose and 44.5% for the full course.

HPV, human papillomavirus; VIA, visual inspection of the cervix with acetic acid.

Available data estimating screening coverage were often based on relatively small samples (noted in Table 3), and age eligibility for screening varied across countries. Some countries without national programs (such as Bhutan, which has had a VIA demonstration project) did offer estimates of CCS coverage, although these data were limited and sometimes based on single studies with small sample sizes. Only one of the countries has an HPV vaccination program, and only Moldova has been supported (in 2017 and 2018) by Gavi for HPV vaccination demonstration projects.

In this study we briefly consider four specific countries of origin as examples on either ends of the spectrum of national cervical cancer control programs. As examples of countries where programs are emerging or not yet in existence, Afghanistan and Democratic Republic (DR) of the Congo are represented by ∼25% of cases (Tables 1 and 2). Afghanistan's National Reproductive Health Policy initiated the country's cervical cancer diagnosis and treatment plan in 2016, and the country is still on the cusp of widespread implementation.^11^ The initial goal included implementing VIA, although the HPV Centre reports that the main test used is cytology for women 15–49 years old with a screening interval of every 5 years. In Afghanistan, there are no estimates of coverage for CCS, and no national HPV immunization program. DR Congo has no national program for primary or secondary prevention.^8^

As examples of countries with extant programs, Colombia and El Salvador are represented by only 2–3% of cases in our samples. Still, data suggest that population coverage is relatively high, between 67% and 70%, while having an opportunistic screening program just like the United States. Only Colombia offers HPV vaccination; El Salvador does not.

## Conclusions

Examining cervical cancer control practices from these 15 countries, it becomes clear that countries of origin for refugee women may lack effective national cervical cancer control programs, which includes both primary prevention of cervical cancer by HPV vaccination and secondary prevention through cervical screening. Furthermore, given the lack of HPV genotyping in all countries but Colombia, it is possible that any screening in their country of origin was inadequate, given that high-performing HPV testing, alone or with cytology, is more effective in identifying precancerous lesions and invasive cancers than screening with cytology alone or by VIA.^12^

When we considered four specific countries of origin, we see examples of the spectrum of possibilities: for the largest groups of refugees resettling in the United States, cervical cancer control in countries of origin (e.g., Afghanistan and DR Congo) is generally poor. In contrast, countries of origin with more robust programs (e.g., Colombia and El Salvador) are represented by small number of women resettling in the United States.

The WHO's ambitious goal to eliminate cervical cancer as a public health problem by 2030 includes three necessary pillars of cancer control: prevention, screening, and treatment.^6^ The specific global goals to achieve elimination include 90% coverage of HPV vaccination of girls by 15 years of age, and 70% coverage of screening with high-performance tests by the ages of 35 and 45 years. This study uses country of origin data that focuses on the first two pillars, prevention and screening, to demonstrate how and why cervical cancer control represents a persistent source of health inequity for refugee women in the United States.

Although high-income countries such as the United States are close to these goals for the overall population, to improve health equity in cervical cancer control, we must pay focused attention to women who migrate and resettle here because they represent a vulnerable and underscreened group.

Individuals who resettle as refugees benefit from relationships with health care providers who are supportive, can anticipate unique needs, and can provide clear culturally responsive information about disease prevention and early detection guidelines, including those related to cervical cancer control.^13^ Individual knowledge, attitudes, and beliefs about health and health care are also situated in a particular cultural context that is informed by country of origin.

As providers caring for these women, our responsibility is to recognize the existence of the global disparity in cervical cancer control and be responsive to this to improve health equity.^14,15^ We can do this by assessing our migrant and refugee patients' need for culturally tailored education around prevention and screening for cervical cancer, and by continuing to research novel ways of increasing screening for this vulnerable population.^16,17^ Although this brief report focuses only on cervical cancer control, it may also serve as a bellwether for increased awareness about disparities related to other cancers and noncommunicable diseases for refugee populations.

## Abbreviations Used

CCS: cervical cancer screening
DR Congo: Democratic Republic of the Congo
FY: fiscal year
HPV: human papillomavirus
IARC: International Agency for Research on Cancer
ICO: Catalan Institute of Oncology
LMIC: low and middle income country
VIA: visual inspection of the cervix with acetic acid
WHO: World Health Organization
